# Endothelial Cell‐Derived Lactate Triggers Bone Mesenchymal Stem Cell Histone Lactylation to Attenuate Osteoporosis

**DOI:** 10.1002/advs.202301300

**Published:** 2023-09-26

**Authors:** Jinhui Wu, Miao Hu, Heng Jiang, Jun Ma, Chong Xie, Zheng Zhang, Xin Zhou, Jianquan Zhao, Zhengbo Tao, Yichen Meng, Zhuyun Cai, Tengfei Song, Chenglin Zhang, Rui Gao, Chang Cai, Hongyuan Song, Yang Gao, Tao Lin, Ce Wang, Xuhui Zhou

**Affiliations:** ^1^ Department of Orthopedics Changzheng Hospital Naval Medical University Shanghai 200003 P. R. China; ^2^ Department of Orthopedics General Hospital of Southern Theatre Command of PLA Guangzhou 510010 P. R. China; ^3^ Department of Orthopedics Shanghai General Hospital Shanghai Jiao Tong University School of Medicine Shanghai 200080 P. R. China; ^4^ Department of Neurology Renji Hospital Shanghai Jiaotong University School of Medicine Shanghai 200127 P. R. China; ^5^ Department of Ophthalmology Changhai Hospital Shanghai 200433 P. R. China; ^6^ Senior Department of Orthopedics The Fourth Medical Center of PLA General Hospital Beijing 100048 P. R. China

**Keywords:** histone lactylation, lactate, metabolic reprogramming, osteoporosis, vascular metabolism

## Abstract

Blood vessels play a role in osteogenesis and osteoporosis; however, the role of vascular metabolism in these processes remains unclear. The present study finds that ovariectomized mice exhibit reduced blood vessel density in the bone and reduced expression of the endothelial glycolytic regulator pyruvate kinase M2 (PKM2). Endothelial cell (EC)‐specific deletion of *Pkm2* impairs osteogenesis and worsens osteoporosis in mice. This is attributed to the impaired ability of bone mesenchymal stem cells (BMSCs) to differentiate into osteoblasts. Mechanistically, EC‐specific deletion of *Pkm2* reduces serum lactate levels secreted by ECs, which affect histone lactylation in BMSCs. Using joint CUT&Tag and RNA sequencing analyses, collagen type I alpha 2 chain (COL1A2), cartilage oligomeric matrix protein (COMP), ectonucleotide pyrophosphatase/phosphodiesterase 1 (ENPP1), and transcription factor 7 like 2 (TCF7L2) as osteogenic genes regulated by histone H3K18la lactylation are identified. PKM2 overexpression in ECs, lactate addition, and exercise restore the phenotype of endothelial PKM2‐deficient mice. Furthermore, serum metabolomics indicate that patients with osteoporosis have relatively low lactate levels. Additionally, histone lactylation and related osteogenic genes of BMSCs are downregulated in patients with osteoporosis. In conclusion, glycolysis in ECs fuels BMSC differentiation into osteoblasts through histone lactylation, and exercise partially ameliorates osteoporosis by increasing serum lactate levels.

## Graphical abstract

The glycolysis of vascular endothelial cells (ECs) fuels the differentiation of BMSCs into osteoblasts through lactate‐mediated histone lactylation, and exercise partially ameliorates osteoporosis through increasing serum lactate. The present study highlights the importance of ECs‐derived lactate in osteogenesis and osteoporosis, while also laying the foundation for the treatment of osteoporosis by targeting vascular metabolism.

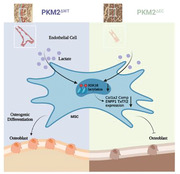

## 1 Introduction

Precise control of osteoblast bone formation and osteoclast bone resorption helps sustain healthy skeletal remodeling.^[^ ^1^ ^]^ Impaired osteoblastic and excessive osteoclastic activities occur among the elderly and women who have reached menopause, eventually leading to osteoporosis.^[^ ^2^ ^]^ Increased bone fragility in osteoporosis causes bone fractures, which are associated with increased mortality and healthcare costs.^[^ ^2^, ^3^ ^]^ Bone marrow mesenchymal stem cells (BMSCs) are the major source of osteoblasts, and aging and menopause often disrupt their differentiation into osteoblasts.^[^ ^4^ ^]^ Blood vessels mediate the transport of oxygen, nutrients, growth factors, and metabolites, which contribute to osteogenesis.^[^ ^5^ ^]^ Blood vessels, particularly the type H vessels, affect BMSC differentiation and osteogenesis.^[^ ^6^ ^]^ A decrease in type H vessels (positive for CD31 and endomucin) is associated with age‐induced osteoporosis. However, total bone blood vessel density is not defined.^[^ ^6a,b^ ^]^ Furthermore, the role of vascular endothelial cell metabolism in osteogenesis and osteoporosis remains unknown.

Vascular endothelial cells (ECs) cover the inner layer of the vasculature and regulate the metabolism of other tissues by controlling oxygen and nutrient supply through angiogenesis.^[^ ^7^ ^]^ In contrast to other cells, ECs produce most of the adenosine triphosphate (ATP) via glycolysis, and angiogenic stimulation further upregulates EC glycolysis to sustain angiogenesis.^[^ ^8^ ^]^ ECs prefer aerobic glycolysis, regardless of sufficient oxygen levels in the blood, and convert most of the glucose to lactate.^[^ ^9^ ^]^ Large amounts of EC‐derived lactate are secreted into the blood and circulated throughout the body. Recent studies have indicated that EC‐derived lactate regulates the angiogenic phenotype of macrophages/microglia in pathological retinopathy and stimulates the pro‐regenerative M2‐like phenotype of macrophages to control muscle regeneration.^[^ ^10^ ^]^ In addition, EC‐derived lactate fuels brain pericytes to maintain the blood–brain barrier function.^[^ ^11^ ^]^ High‐intensity exercise performed at regular intervals produces large amounts of lactate, which induces cerebral angiogenesis.^[^ ^12^ ^]^ These studies suggest that EC‐derived lactates function as new signaling molecules in developmental and pathological conditions.

The lactate‐derived lactylation of histone lysine residues serves as an epigenetic modification that stimulates gene transcription.^[^ ^13^ ^]^ Histone lactylation in mouse osteoblast precursor cell lines increases during osteoblast differentiation, indicating that lactylation plays a role in osteogenesis.^[^ ^14^ ^]^ Pyruvate kinase catalyzes the final rate‐limiting step of glycolysis, thus generating ATP and pyruvate, which are then converted to lactate by lactate dehydrogenase A (LDHA).^[^ ^15^ ^]^ Specific deletion of the M2 isoform of pyruvate kinase (*Pkm2*) in microglia reduces lactate production and disrupts histone lactylation.^[^ ^16^ ^]^ ECs express PKM2 exclusively over the M1 isoform of pyruvate kinase (PKM1).^[^ ^17^ ^]^ Although specific deletion of *Pkm2* in ECs suppresses retinal blood vessel growth, the role of endothelial PKM2 in skeletal angiogenesis and bone remodeling remains largely unknown.^[^ ^17^ ^]^

The ovariectomy‐induced osteoporosis model (ovariectomized mice; OVX mice) is widely used to study postmenopausal osteoporosis.^[^ ^18^ ^]^ However, the blood vessel phenotype of OVX mice remains elusive. In the present study, we investigated the metabolic crosstalk between ECs and BMSCs and found that ECs use their glycolytic capacity to promote osteogenesis. In OVX model, both the density of bone blood vessels and PKM2 expression in ECs decreased significantly. Specific deletion of *Pkm2* (Pkm2^ΔEC^) in ECs impaired osteogenesis and worsened the phenotype of osteoporosis in OVX mice. PKM2 overexpression in ECs, addition of lactate, and high‐intensity interval exercise restored the phenotype of PKM2‐deficient mice. Mechanistically, EC‐derived lactate increased histone lactylation in BMSCs. CUT&Tag and RNA‐sequencing analyses revealed that H3K18la lactylation directly stimulated the transcription of osteogenic collagen type I alpha 2 chain (COL1A2), cartilage oligomeric matrix protein (COMP), ectonucleotide pyrophosphatase/phosphodiesterase 1 (ENPP1), and transcription factor 7 like 2 (TCF7L2). Moreover, serum metabolomics indicated that patients with osteoporosis had relatively low lactate levels. Additionally, histone lactylation and related osteogenic genes of BMSCs were downregulated in patients with osteoporosis. These data suggest that EC‐derived lactate triggers histone lactylation in BMSCs to attenuate osteoporosis.

## 2 Results

### 2.1 Bone Blood Vessels and Endothelial PKM2 Decreased in OVX Mice

**Figure 1:**
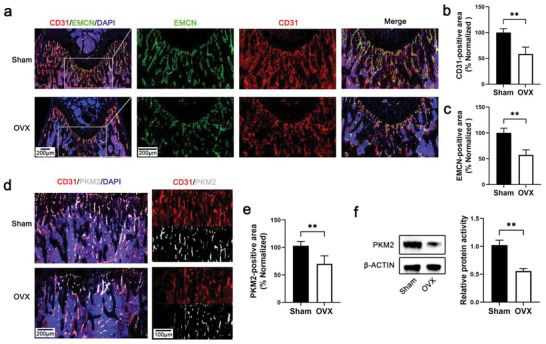
Bone blood vessels and endothelial PKM2 decreased in OVX mice. a) Representative confocal images of sham and OVX mice femurs stained with CD31 (Red) or EMCN (Green) and DAPI (blue) (scale bar 200 µm). b) Quantification of CD31‐positive vessel area in the BM cavity of the femur sections. *n* = 4 mice (**p < 0.01). c) Quantification of EMCN‐positive vessel area in the BM cavity of the femur sections. *n* = 4 mice (**p < 0.01). d) Representative confocal images of sham and OVX mice femurs stained with CD31 (Red), PKM2 (white), and DAPI (blue) (scale bars: 200 µm and 100 µm). e) Quantification of PKM2‐positive area in the BM cavity of the femur sections. *n* = 4 mice (**p < 0.01). f) The expression of PKM2 in BMSCs sorted from the tibiae and femurs of mice. *n* = 3 mice (**p < 0.01).

The OVX‐induced osteoporosis model was constructed as described previously.^[^ ^19^ ^]^ Hematoxylin and eosin (H&E) staining showed apparent trabecular bone loss in OVX mice (Figure S1a, Supporting Information). Micro‐computed tomography (micro‐CT) further indicated reduced bone mineral density (BMD) and bone volume/total volume (BV/TV) in the distal femur (Figure S1b–d, Supporting Information). Both total bone blood vessel and type H vessel density decreased in OVX mice (**Figure** **1**a–c). In this study, we examined the expression of the final rate‐limiting glycolytic enzyme PKM2 in the bone blood vessels of OVX mice. PKM2 was mainly expressed in the blood vessels of the bone, which may be attributed to the high glycolytic properties of vascular endothelial cells (Figure 1d). The results showed a significantly decreased expression of PKM2 in the bone ECs of OVX mice (Figure 1d,e). Western blot analysis of magnet‐activated cell sorting (MACS) bone marrow endothelial cells (BMECs) showed attenuated PKM2 expression in OVX mice (Figure 1f). Collectively, these data suggest that decreased endothelial PKM2 expression contributes to postmenopausal osteoporosis.

### 2.2 Endothelial PKM2 Controls Bone Blood Vessel Density and Osteogenesis

**Figure 2:**
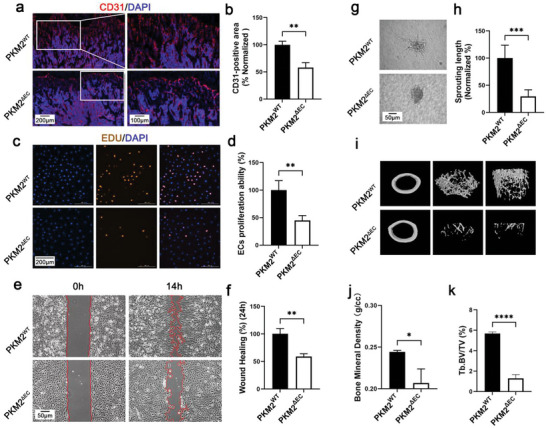
Ablation of endothelial PKM2 decreases bone blood vessels and osteogenesis. a) Representative confocal images of femurs stained with CD31 (Red) and DAPI (blue) (scale bars: 200 µm and 100 µm). b) Quantification of CD31‐positive vessel area in the BM cavity of the femur sections. *n* = 3 mice (**p < 0.01). c) Cell proliferation determined by EdU staining. EdU (Red), DAPI (Blue) (scale bar: 200 µm). d) Statistical result for cell proliferation. *n* = 4 (**p < 0.01). e) Representative images of migrated cells (scale bar: 50 µm). f) Statistical result for cell migration. *n* = 4 (**p < 0.01). g) Representative images of cell sprouting (scale bar: 50 µm). h) Statistical result for cell sprouting. *n* = 4 (***p < 0.001). i) Representative micro‐CT images of the distal femur in *Pkm2* ^ΔEC^ mice. j,k) Quantitative analysis of trabecular bone volume per tissue volume (BV/TV) and bone mineral density (BMD) in *Pkm2* ^ΔEC^ mice. *n* = 3 mice (*p < 0.05 and ****p < 0.0001).

To study the role of PKM2 in bone endothelium, *Pkm2* ^floxed/floxed^ (*Pkm2* ^fl/fl^) mice were used and intercrossed with Cdh5‐Cre^Ert2^ transgenic mice to generate endothelial *Pkm2*‐deleted mice (*Pkm2* ^ΔEC^) (Figure S2a, Supporting Information). *Pkm2* ^floxed/floxed^ mice with negative Cdh5‐Cre^Ert2^ were used as controls (Pkm2^wt^). Western blot analysis of MACS‐sorted tibial BMECs showed an apparent reduction in the expression of PKM2 in 4 weeks old *Pkm2* ^ΔEC^ mice compared to that in control littermates (Figure S2b,c, Supporting Information). Using Seahorse Flux analysis, we evaluated the glycolytic function of BMECs from *Pkm2* ^ΔEC^ mice (BMECs^Δpkm2^) by measuring their extracellular acidification rate (ECAR). The glycolytic flux in BMECs^Δpkm2^ decreased significantly compared with that in BMECs from *Pkm2* ^wt^ mice (BMECs^wt^) (Figure S2d,e, Supporting Information). Immunofluorescence analysis showed that EC‐specific deletion of *Pkm2* significantly reduced bone blood‐vessel density in 4 weeks old mice (**Figure** **2**a,b). Additionally, type H vessel density was substantially reduced in *Pkm2* ^ΔEC^ mice (Figure S2f,g, Supporting Information). Furthermore, we evaluated the functions of BMECs isolated from the tibiae and femurs of the mice. The results showed that BMECs^Δpkm2^ exhibited impaired proliferation, migration, and tube formation compared with BMECs^wt^ (Figure 2c–h). Micro‐CT data revealed that BMD and BV/TV of the distal femur decreased in 4 weeks old *Pkm2* ^ΔEC^ mice compared to that in control littermates (Figure 2i–k). These data indicate an important role of endothelial PKM2‐mediated glycolysis in osteogenesis.

### 2.3 Deletion of Endothelial PKM2 Worsens Osteoporosis

**Figure 3:**
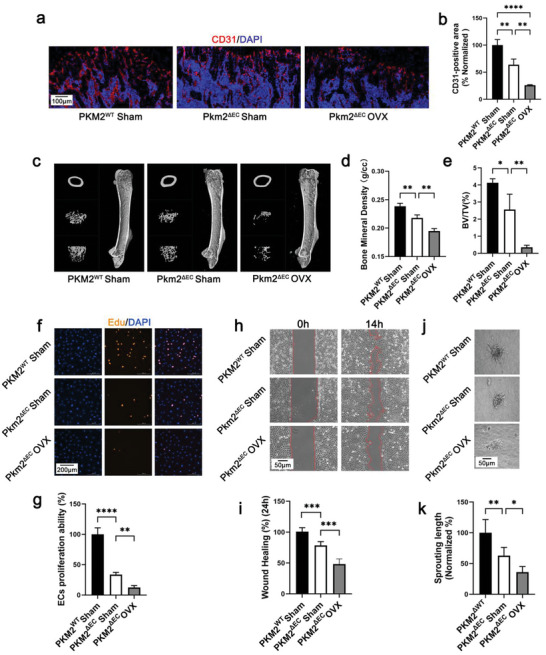
Ablation of endothelial PKM2 worsens osteoporosis. a) Representative confocal images of femurs stained with CD31 (Red) and DAPI (blue) (scale bar 100 µm). b) Quantification of CD31‐positive vessel area in the BM cavity of the femur sections. *n* = 5 mice (**p < 0.01). c) Representative micro‐CT images of the distal femur in *Pkm2* ^wt^ mice, *Pkm2* ^ΔEC^ mice, and OVX *Pkm2* ^ΔEC^ mice. d,e) Quantitative analysis of trabecular bone volume per tissue volume (BV/TV) and bone mineral density (BMD) in *Pkm2* ^wt^ mice, *Pkm2* ^ΔEC^ mice, and OVX *Pkm2* ^ΔEC^ mice. *n* = 5 mice (**p < 0.05 and **p < 0.01). f) Cell proliferation determined by EdU staining. EdU (Red), DAPI (Blue) (scale bar: 200 µm). g) Statistical result for cell proliferation. *n* = 4 (**p < 0.01 and ***p < 0.001). h) Representative images of migrated cells (scale bar: 50 µm). i) Statistical result of cell migration. *n* = 4 (***p < 0.001). j) Representative images for cell sprouting (scale bar: 50 µm). k) Statistical result for cell sprouting. *n* = 4 (*p < 0.05 and **p < 0.01).

To study the role of endothelial PKM2 in pathological conditions, *Pkm2* ^ΔEC^ mice were used for generating OVX mice. The data indicated that the total bone blood vessel density and type H vessel density decreased in OVX mice, and EC‐specific deletion of *Pkm2* worsened this phenotype (**Figure** **3**a,b; Figure S3a,b, Supporting Information). Bone density was evaluated using micro‐CT; compared with OVX *Pkm2* ^wt^ mice, OVX *Pkm2* ^ΔEC^ mice showed a decrease in the BMD and BV/TV of the distal femur (Figure 3c–e). Consistent results were obtained by H&E staining, which showed that the trabecular bone loss worsened in OVX *Pkm2* ^ΔEC^ mice (Figure S3c, Supporting Information). Additionally, we evaluated the angiogenic properties of BMECs from *Pkm2* ^wt^ mice, OVX *Pkm2* ^wt^ mice, and OVX *Pkm2* ^ΔEC^ mice. BMECs from OVX *Pkm2* ^wt^ mice exhibited impaired proliferation, migration, and tube formation compared to those from *Pkm2* ^wt^ mice (Figure 3f–k). BMECs from OVX *Pkm2* ^ΔEC^ mice showed a greater reduction in proliferation, migration, and tube‐formation abilities compared with BMECs sorted from OVX *Pkm2* ^wt^ mice (Figure 3f–k). The results confirmed the important role of endothelial PKM2 in osteogenesis and osteoporosis.

### 2.4 Endothelial Lactate Affects the Differentiation of BMSCs to Osteoblasts

**Figure 4:**
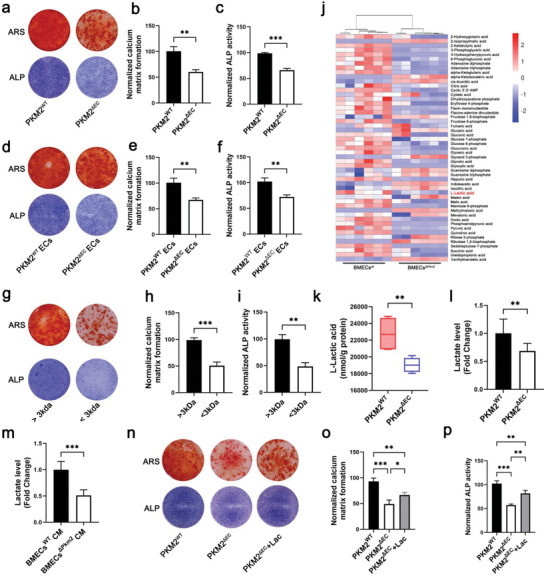
Lactate mediates the crosstalk between BMECs and BMSCs. a) Representative images of ARS and ALP staining of isolated BMSCs from *Pkm2* ^ΔEC^ mice and *Pkm2* ^wt^ mice. b,c) Quantitative analysis of ARS and ALP staining of isolated BMSCs from *Pkm2* ^ΔEC^ mice and *Pkm2* ^wt^ mice. *n* = 4 (**p < 0.01 and ***p < 0.001). d) Representative images of ARS and ALP staining of BMSCs co‐cultured with BMECs^Δpkm2^ and BMECs^wt^. e,f) Quantitative analysis of ARS and ALP staining of BMSCs co‐cultured with BMECs^Δpkm2^ and BMECs^wt^. *n* = 4 (**p < 0.01). g) Representative images of ARS and ALP staining of BMSCs treated with conditioned cell culture medium fractions <3 kDa. h,i) Quantitative analysis of ARS and ALP staining of BMSCs treated with fractions <3 kDa from conditioned cell‐culture medium. *n* = 4 (**p < 0.01 and ***p < 0.001). j) Heatmap of the metabolites from BMECs^Δpkm2^ and BMECs^wt^ using targeted metabolomics. k) The concentration of lactate from BMECs^Δpkm2^ and BMECs^wt^ determined by targeted metabolomic. l) Decreased levels of lactate in BMECs^Δpkm2^ determined by lactic acid test kit (**p < 0.01). m) Decreased levels of lactate in the conditioned medium of BMECs^Δpkm2^. *n* = 4 (***p < 0.001). n) Representative images of ARS and ALP staining of lactate‐treated BMSCs. o,p) Quantitative analysis of ARS and ALP staining of isolated BMSCs treated with lactate. *n* = 4 (*p < 0.05, **p < 0.01, and ***p < 0.001).

Recent studies have indicated that bone blood vessels affect osteogenesis through crosstalk with BMSCs.^[^ ^4^, ^6^ ^]^ To clarify whether the EC‐specific deletion of *Pkm2* in mice impairs the differentiation of BMSCs, we isolated BMSCs from *Pkm2* ^ΔEC^ and *Pkm2* ^wt^ mice. The data showed that BMSCs from *Pkm2* ^ΔEC^ mice exhibited a reduced ability to differentiate into osteoblasts, according to Alizarin Red S (ARS) and alkaline phosphatase (ALP) staining (**Figure** **4**a–c). To confirm the crosstalk between ECs and BMSCs, we sorted BMECs from *Pkm2* ^ΔEC^ mice and *Pkm2* ^wt^ mice and co‐cultured them with BMSCs (isolated from wild‐type mice). BMSCs co‐cultured with BMECs^Δpkm2^ showed decreased ARS and ALP staining (Figure 4d–f). Furthermore, we used PKM2 siRNAs to knockdown PKM2 expression in BMECs and co‐cultured these BMECs with BMSCs. The result was consistent with that for BMECs^Δpkm2^ (Figure S4a–c, Supporting Information). Similar results were obtained using the conditioned cell culture medium from BMECs^Δpkm2^ and PKM2‐siRNA‐treated BMECs (Figure S4d–i, Supporting Information). PKM2 is a key glycolytic regulator in ECs, and BMECs^Δpkm2^ exhibited suppressed glycolytic flux compared with BMECs^wt^ (Figure S2d,e, Supporting Information). These results suggest that EC glycolysis disruption impaired the function of BMSCs; however, the specific factors underlying this effect needed to be clarified.

To better identify soluble factors in the conditioned medium, 3K centrifugal filters were used for fractionating the conditioned cell culture medium according to size (<3 and >3 kDa), and both fractions were tested for their activity. BMSCs treated with the <3 kDa fraction from the BMECs^Δpkm2^ medium exhibited reduced ARS and ALP staining (Figure 4g–i). However, the >3 kDa fraction from the BMEC^Δpkm2^ medium and that from the BMEC^wt^ medium showed no difference in their effect on BMSCs (Figure S5a–c, Supporting Information). Meanwhile, boiling the conditioned medium did not change its effect on BMSC differentiation (Figure S5d–f, Supporting Information). These results suggest that the factors mediating BMEC crosstalk with BMSCs may be heat stable and likely to be low‐molecular‐weight metabolites. Targeted metabolomics of BMECs^Δpkm2^ and BMECs^wt^ revealed a significant decrease in the intracellular levels of glycolysis‐related metabolites, ATP, phosphoglyceric acid, glucose 6‐phosphate, glucose 1‐phosphate, fructose 6‐phosphate, and pyruvic acid lactate in BMECs^Δpkm2^ (Figure 4j). These data indicated that glycolysis in BMECs^Δpkm2^ was significantly suppressed. Among these metabolites, we focused on lactate because of its role as a signaling molecule in developmental and pathological conditions (Figure 4k). Lactate levels were confirmed using a lactate detection kit (Figure 4l). Therefore, we tested whether lactate affected BMSC differentiation. We found that the lactate levels were lower in the conditioned medium derived from BMECs^Δpkm2^ (Figure 4m). Furthermore, the addition of lactate partially rescued the reduction in ARS and ALP staining in BMSCs co‐cultured with BMECs^Δpkm2^ (Figure 4n–p). Collectively, these data indicate that lactate mediates the crosstalk between BMECs and BMSCs.

### 2.5 BMSCs Histone H3K18la Lactylation Boosts Osteogenic Genes

**Figure 5:**
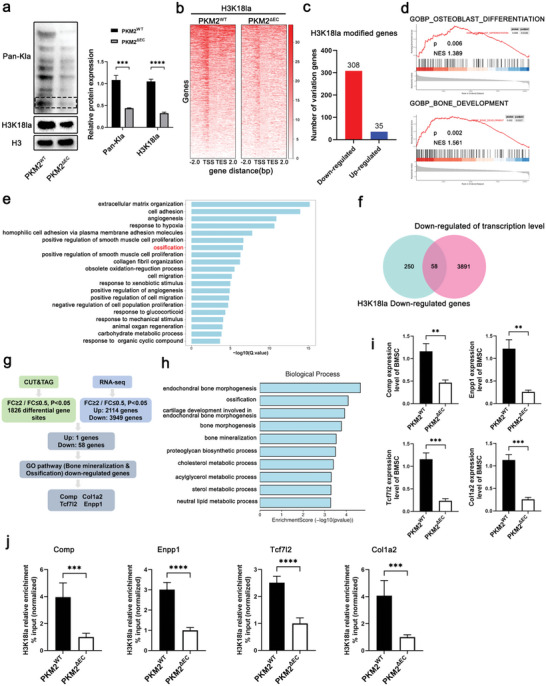
H3K18la lactylation boosts osteogenic genes in BMSCs. a) The levels of pan‐Kla and H3K18la decrease in BMSCs isolated from *Pkm2* ^ΔEC^ mice (***p < 0.001). b) Heatmap for H3K18la binding peaks in BMSCs isolated from *Pkm2* ^ΔEC^ and *Pkm2* ^wt^ mice. The red bar indicates the signal intensity of CUT & Tag. c) H3K18la modified genes that are differently regulated in BMSCs isolated from Pkm2^ΔEC^ and *Pkm2* ^wt^ mice. d) GSEA of H3K18la‐bound genes in BMSCs isolated from *Pkm2* ^ΔEC^ and *Pkm2* ^wt^ mice. e) Gene ontology analysis of top 20 ontology terms. f,g) Joint CUT&Tag and RNA‐seq analysis identify COL1A2, COMP, ENPP1, and TCF7L2 as downstream targets of H3K18la. h) Joint CUT&Tag and RNA‐seq analysis show bone morphogenesis, ossification, and bone mineralization were mainly affected. i) qPCR analysis of COL1A2, COMP, ENPP1, and TCF7L2 in BMSCs isolated from *Pkm2* ^ΔEC^ and *Pkm2* ^wt^ mice. *n* = 4 (**p < 0.01 and ***p < 0.001). j) ChIP‐qPCR analysis of H3K18la binding to the promoter regions of COL1A2, COMP, ENPP1, and TCF7L2 in BMSCs isolated from *Pkm2* ^ΔEC^ and *Pkm2* ^wt^ mice. *n* = 4 (***p < 0.001 and ****p < 0.0001).

Lactate‐derived lactylation of histone lysine residues facilitates gene transcription.^[^ ^13a^ ^]^ Therefore, the lactylation of BMSCs from *Pkm2* ^ΔEC^ mice and *Pkm2* ^wt^ mice was evaluated. Suppression of histone lactylation was detected in the BMSCs from *Pkm2* ^ΔEC^ mice (**Figure** **5**a). In particular, H3K18la histone lactylation was affected (Figure 5a). Furthermore, we detected changes in other histone lactylation sites. H3K4la and H4K12la showed no difference between BMSCs from *Pkm2* ^ΔEC^ mice and *Pkm2* ^Δpkm2^ mice (Figure S6, Supporting Information). However, H4K5la expression was slightly decreased in BMSCs from *Pkm2* ^ΔEC^ mice; nevertheless, this decrease was considerably lower than the decrease in H3K18la expression (Figure S6, Supporting Information). Therefore, we supposed that H3K18la plays a major role in BMSC differentiation. To identify candidate target genes regulated by H3K18la histone lactylation, we performed a CUT&Tag assay using an H3K18la antibody. The results showed that H3K18la was enriched in the promoter and upstream gene regions (Figure 5b; Figure S7a, Supporting Information). BMSCs from *Pkm2* ^ΔEC^ mice showed 308 downregulated and 35 upregulated genes with differential H3K18la modifications (Figure 5c). KEGG analysis showed that H3K18la‐modified genes were involved in the development, energy metabolism, endocrine system, and lipid metabolism (Figure S7b, Supporting Information). Furthermore, Gene Set Enrichment Analysis (GSEA) of H3K18la‐bound genes showed significant enrichment in “osteoblast differentiation” and “bone development” (Figure 5d). To clarify the target genes of H3K18la, we further performed RNA sequencing of BMSCs isolated from *Pkm2* ^ΔEC^ mice and *Pkm2* ^wt^ mice; 3949 downregulated genes and 2114 upregulated genes were detected in BMSCs from *Pkm2* ^ΔEC^ mice (Figure S7c, Supporting Information). Gene ontology (GO) analysis showed that ossification belonged to the top 20 affected categories (Figure 5e). Joint CUT&Tag and RNA‐seq analyses revealed 58 down‐regulated genes in BMSCs from *Pkm2* ^ΔEC^ mice (Figure 5f,g; Figure S7d, Supporting Information). These genes are involved in bone morphogenesis, ossification, and bone mineralization (Figure 5h). COL1A2, COMP, ENPP1, and TCF7L2 were identified as the potential target genes of H3K18la lactylation (Figure 5g). A genomic snapshot revealed H3K18la modification sites in COL1A2, COMP, ENPP1, and TCF7L2 (Figure S7e, Supporting Information). ChIP‐qPCR and RT‐qPCR confirmed the regulation of COL1A2, COMP, ENPP1, and TCF7L2 expression by H3K18la lactylation (Figure 5i,j). We identified in BMSCs the target genes of H3K18la that contribute to osteoblast differentiation.

### 2.6 Endothelial Lactate Controls Differentiation of BMSCs to Osteoblasts through H3K18la Lactylation

**Figure 6:**
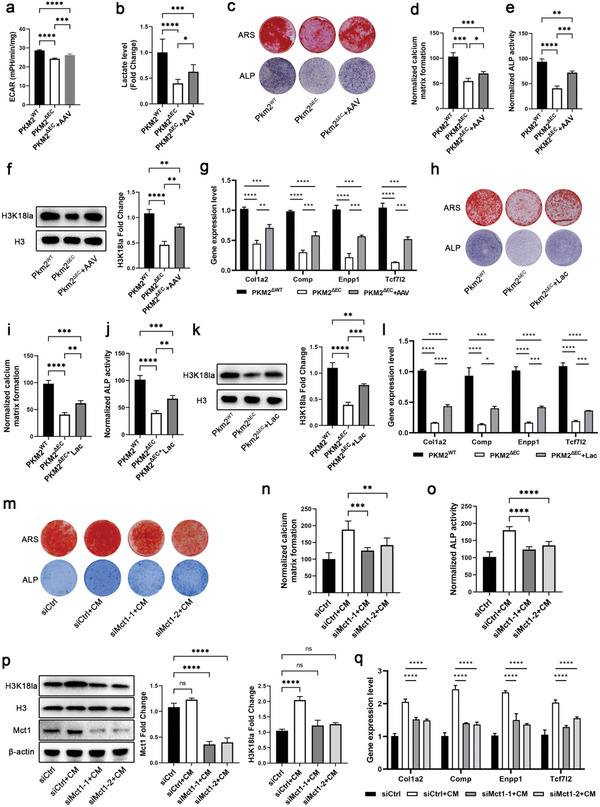
Endothelial lactate contributes to BMSC differentiation toward osteoblasts. a) Overexpression of PKM2 in ECs increases the glycolytic flux. *n* = 3 (***p < 0.001 and ****p < 0.0001). b) Overexpression of PKM2 in ECs increases lactate production. *n* = 3 (*p < 0.05, ***p < 0.001, and ****p < 0.0001). c) Representative images of ARS and ALP staining of BMSCs co‐cultured with ECs infected with PKM2 adenovirus. d,e) Quantitative analysis of ARS and ALP staining of BMSCs co‐cultured with ECs infected with PKM2 adenovirus. *n* = 4 (*p < 0.05, **p < 0.01, ***p < 0.001, and ****p < 0.0001). f) Histone H3K18la lactylation increases in BMSCs co‐cultured with ECs infected with PKM2 adenovirus (**p < 0.01 and ****p < 0.0001). g) qPCR analysis of COL1A2, COMP, ENPP1, and TCF7L2 in BMSCs co‐cultured with ECs infected with PKM2 adenovirus. *n* = 3 (**p < 0.01, ***p < 0.001, and ****p < 0.0001). h) Representative images of ARS and ALP staining of BMSCs cultured in a conditioned medium supplemented with lactate. i,j) Quantitative analysis of ARS and ALP staining of BMSCs cultured in a conditioned medium supplemented with lactate. *n* = 4 (**p < 0.01, ***p < 0.001, and ****p < 0.0001). k) Histone H3K18la lactylation increased in BMSCs cultured in conditioned medium supplemented with lactate (**p < 0.01, ***p < 0.001, and ****p < 0.0001). l) qPCR analysis of COL1A2, COMP, ENPP1, and TCF7L2 in BMSCs cultured in conditioned medium supplemented with lactate. *n* = 4 (*p < 0.05, ***p < 0.001, and ****p < 0.0001). m) Representative images of ARS and ALP staining of BMSCs treated with MCT1 siRNAs in a conditioned medium. n,o) Quantitative analysis of ARS and ALP staining of BMSCs treated with MCT1 siRNAs in a conditioned medium. *n* = 4 (**p < 0.01, ***p < 0.001, and ****p < 0.0001). p) Histone H3K18la lactylation decreases in BMSCs treated with MCT1 siRNAs in conditioned medium (****p < 0.0001). q) qPCR analysis of COL1A2, COMP, ENPP1, and TCF7L2 in BMSCs treated with MCT1 siRNAs in conditioned medium supplemented with lactate. *n* = 4 (****p < 0.0001).

To understand the role of BMSC H3K18la lactylation in osteogenesis, we generated a vascular‐targeting PKM2 adenovirus to rescue the expression of endothelial PKM2 in vitro. Serotype 9 adeno‐associated virus (AAV9) was used for carrying *Pkm2* cDNA. The efficiency of the PKM2 adenovirus was confirmed using western blotting and qPCR (Figure S8a,b, Supporting Information). PKM2 overexpression in BMECs^Δpkm2^ rescued the reduction in cell proliferation, migration, and tube formation (Figure S8c–h, Supporting Information). Meanwhile, the decrease in glycolytic flux and lactate levels in BMECs^Δpkm2^ were rescued by the PKM2 adenovirus (**Figure** **6**a,b). BMECs^Δpkm2^ infected with PKM2 adenovirus were co‐cultured with BMSCs, which improved the decrease in ARS and ALP staining in the BMEC^Δpkm2^ co‐cultured group (Figure 6c–e). H3K18la lactylation and target genes were evaluated. PKM2 overexpression in BMECs^Δpkm2^ increased histone H3K18la lactylation in BMSCs when co‐cultured (Figure 6f). Consistent results were obtained for the expression and H3K18la‐regulation of target genes (Figure S8i, Supporting Information). Conditioned medium from BMECs^Δpkm2^ infected with the PKM2 adenovirus showed increased histone H3K18la lactylation and target gene expression compared with the medium from BMECs^Δpkm2^ (Figure S9a,b, Supporting Information).

We evaluated the effect of lactate on BMSCs to determine whether lactate could rescue this phenotype in vitro. Lactate increased the expression of H3K18la dose dependently, and a concentration of 5 mm upregulated H3K18la levels apparently (Figure S9c,d, Supporting Information). However, a higher concentration of lactate impaired the viability of BMSCs (Figure S9e, Supporting Information). Considering the lactate concentration used in vitro experiments in previous studies, a concentration of 5 mm was used in our subsequent experiments. The addition of 5 mm lactate to the medium of BMSCs from *Pkm2* ^ΔEC^ mice rescued the decrease in ARS and ALP staining (Figure 6i–k) and upregulated histone H3K18la lactylation (Figure 6l). Similar results were obtained for the expression and H3K18la‐regulation of target genes (Figure S9f, Supporting Information). Furthermore, the addition of lactate to the conditioned cell culture medium from BMECs^Δpkm2^ increased ARS and ALP staining of BMSCs (Figure S9g–i, Supporting Information). Histone H3K18la lactylation and the expression of its target genes were rescued in BMSCs (Figure S9j–l, Supporting Information).

Monocarboxylate transporter‐1 (MCT1) is the main transporter that catalyzes inward lactate movement across the plasma membrane.^[^ ^20^ ^]^ Knockdown of MCT1 expression using siRNAs decreased ARS and ALP staining in BMSCs treated with the conditioned medium of BMECs (Figure 6m–o). Further data indicated that MCT1 siRNA suppressed H3K18la levels and target gene expression (Figure 6p,q). To better understand the role of histone lactylation in osteogenic gene expression, we evaluated the effects of A485, a p300 inhibitor, on BMSCs. p300 is a well‐known acylation modification writer, and the deletion or inhibition of p300 decreased Kla levels. A485 treatment decreased ARS and ALP staining in BMSCs treated with conditioned medium from BMECs (Figure S10a–c, Supporting Information). Additionally, it decreased H3K18la levels and suppressed the expression of target genes in BMSCs treated with a conditioned medium derived from BMECs (Figure S10d,e, Supporting Information). In summary, restoration of EC glycolysis or lactate levels rescued the differentiation of BMSCs into osteoblasts.

### 2.7 Increasing Lactate Levels Improve Bone Mineral Density in Mice

**Figure 7:**
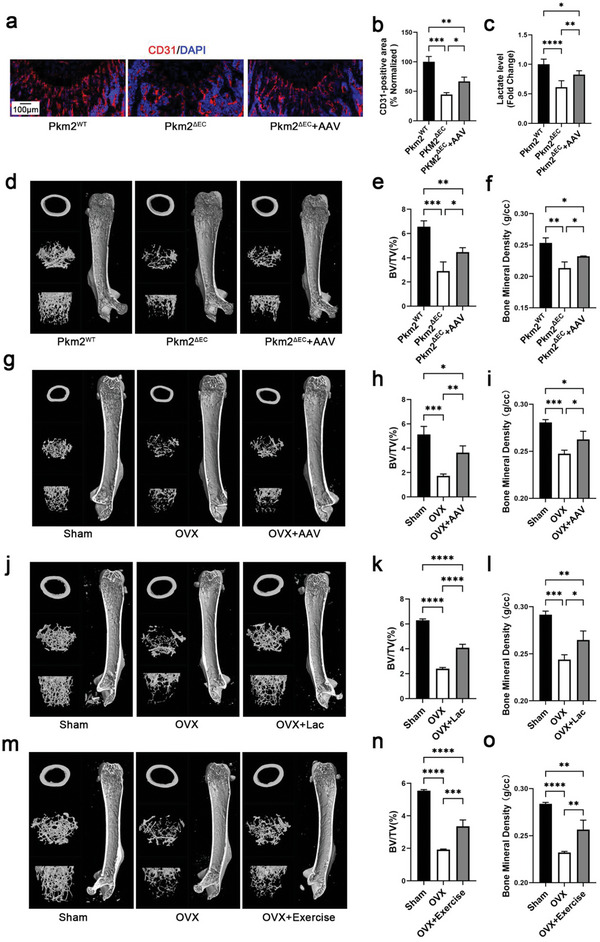
Endothelial lactate restores the phenotype of PKM2‐deficient mice and improves osteoporosis. a) Representative confocal images of femurs stained with CD31 (Red) and DAPI (blue) of *Pkm2* ^wt^ mice, *Pkm2* ^ΔEC^ mice, and *Pkm2* ^ΔEC^ mice treated with PKM2 adenovirus. Scale bars, 100 µm. b) Quantification of CD31‐positive vessel area in the BM cavity of the femur sections. *n* = 4 mice (*p < 0.05, **p < 0.01, and ***p < 0.001). c) PKM2 adenovirus treatment increases serum lactate in *Pkm2* ^ΔEC^ mice. *n* = 4 mice (*p < 0.05, **p < 0.01, and ****p < 0.0001). d) Representative micro‐CT images of the distal femur in *Pkm2* ^wt^ mice, *Pkm2* ^ΔEC^ mice, and *Pkm2* ^ΔEC^ mice treated with PKM2 adenovirus. e,f) Quantitative analysis of trabecular bone volume per tissue volume (BV/TV) and bone mineral density (BMD). *n* = 5 mice (*p < 0.05, **p < 0.01, and ***p < 0.001). g) Representative micro‐CT images of the distal femur in Sham, OVX, and OVX mice treated with PKM2 adenovirus. h,i) Quantitative analysis of trabecular BV/TV and BMD. *n* = 4 mice (*p < 0.05, **p < 0.01, and ***p < 0.001). j) Representative micro‐CT images of the distal femur in Sham, OVX, and OVX mice treated with lactate. k,l) Quantitative analysis of trabecular BV/TV and BMD. *n* = 4 mice (*p < 0.05, **p < 0.01, ***p < 0.001, and ****p < 0.0001). m) Representative micro‐CT images of the distal femur in Sham, OVX, and exercised OVX mice. n,o) Quantitative analysis of trabecular BV/TV and BMD. *n* = 4 mice (**p < 0.01, ***p < 0.001, and ****p < 0.0001).

To determine metabolic crosstalk between ECs and BMSCs, we explored the effects of histone H3K18la lactylation on osteogenesis in vivo. Both total bone vessel density and type H vessel density increased in *Pkm2* ^ΔEC^ mice treated with PKM2 adenovirus compared with that in *Pkm2* ^ΔEC^ mice (**Figure** **7**a,b; Figure S11a,b, Supporting Information). Evaluation of lactate levels showed that PKM2 adenovirus treatment increased serum lactate levels in *Pkm2* ^ΔEC^ mice (Figure 7c). Micro‐CT data revealed that the BMD and BV/TV of the distal femur improved in 4 weeks old *Pkm2* ^ΔEC^ mice treated with PKM2 adenovirus (Figure 7d–f). Similar results were shown by H&E staining (Figure S11c, Supporting Information). Osteoporosis was partially rescued by the PKM2 adenovirus in the bones of OVX mice, as assessed by micro‐CT and H&E staining (Figure 7g–i; Figure S11d, Supporting Information).

Next, we investigated whether the addition of lactate improved osteoporosis in the OVX mouse model. The mice were subcutaneously injected with lactate every 2 days after ovariectomy. Two months later, bone density was evaluated. Both total bone vessel and type H vessel intensity of OVX mice treated with lactate increased compared with that of OVX mice (Figure S11e–g, Supporting Information). Micro‐CT data revealed that the BMD and BV/TV of the distal femur were partially rescued after lactate treatment (Figure 7j–l). H&E staining revealed that trabecular bone loss was lower in the OVX mice treated with lactate than in untreated OVX mice (Figure S12a, Supporting Information). These data highlight the important role of lactate in the crosstalk between ECs and BMSCs.

Exercise has been widely recognized as a therapeutic option for preventing osteoporosis; however, its underlying mechanism remains elusive.^[^ ^21^ ^]^ The results of the present study indicate that exercise‐induced lactate may be beneficial. Our data showed that high‐intensity interval exercise increased blood lactate levels in OVX mice (Figure S12b, Supporting Information). Micro‐CT data revealed that the BMD and BV/TV of the distal femur were partially rescued in OVX mice after exercise (Figure 7m–o). Similar results were obtained using H&E staining (Figure S12c, Supporting Information).

### 2.8 Lactate Levels Were Negatively Correlated with Osteoporosis through BMSC Histone Lactylation

**Figure 8:**
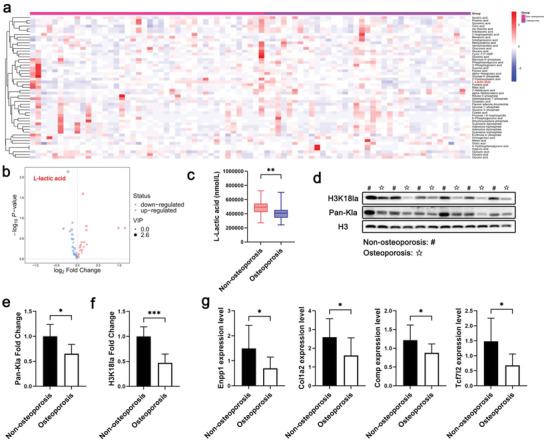
Clinical samples indicate the protective role of lactate in patients with osteoporosis. a) Heatmap of targeted metabolomics of blood from patients. b) Volcano plot presents the upregulated and downregulated metabolites. c) Lactate is downregulated in the osteoporosis group (**p < 0.01). d) Histone pan‐kla is decreased in BMSCs isolated from the bone marrow of patients with osteoporosis. e) Quantitative analysis of pan‐kla in BMSCs isolated from the bone marrow of patients with osteoporosis (*p < 0.05). f) Histone H3K18la lactylation is decreased in BMSCs isolated from the bone marrow of patients with osteoporosis (*p < 0.05). g) Quantitative analysis of histone H3K18la lactylation in BMSCs isolated from the bone marrow of patients with osteoporosis (*p < 0.05). h) qPCR analysis shows decreased levels of COL1A2, COMP, ENPP1, and TCF7L2 in BMSCs isolated from the bone marrow of patients.

To better understand the possible role of lactate in osteoporosis, we identified the metabolites involved in glucose metabolism using targeted metabolomics. We collected 86 serum samples from patients with and those without osteoporosis, according to BMD measured using dual‐energy X‐ray absorptiometry. Age, body mass index, fasting glucose level, and BMD are summarized in Table S1 (Supporting Information). Metabolomic analysis showed that two metabolites in the osteoporosis group were significantly altered (**Figure** **8**a,b). Serum lactate levels decreased in patients with osteoporosis (Figure 8c). These data suggest that lactate level may serve as a negative biomarker for osteoporosis diagnosis and management. Next, we isolated BMSCs from the BM aspirates of patients with osteoporosis and evaluated histone lactylation in human BMSCs. BMSCs isolated from patients with osteoporosis showed decreased histone H3K18la lactylation compared with that from patients without osteoporosis (Figure 8d–f). qPCR analysis of the target genes of H3K18la lactylation indicated that the expression of the osteogenic genes COL1A2, COMP, ENPP1, and TCF7L2 was decreased in BMSCs isolated from patients with osteoporosis compared with that in BMSCs from patients without osteoporosis (Figure 8g).

## 3 Discussion

**Figure 9:**
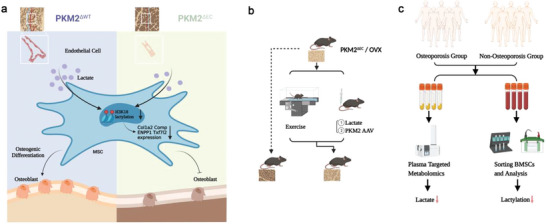
Schematic illustration showing the important role of ECs‐derived lactate in osteogenesis and osteoporosis via histone lactylation. a) ECs‐derived lactate triggers mesenchymal stem cell histone lactylation to control osteoblast differentiation. b) Exercise, addition of lactate, and PKM2 adenovirus improve BMD in OVX mice. c) Lactate levels and BMSC histone lactylation are decreased in the osteoporosis group.

Blood vessels transport oxygen, nutrients, growth factors, and metabolites to maintain vial movement.^[^ ^22^ ^]^ ECs cover the inner wall of the blood vessels, which have recently been found to control osteogenesis.^[^ ^6a^ ^]^ However, the roles of EC metabolism in osteogenesis and osteoporosis are unclear. The present study found that the disruption of EC glycolysis by the specific deletion of *Pkm2* in ECs suppressed osteogenesis and worsened osteoporosis. Interestingly, lactate, a by‐product of glycolysis, played an important role. EC‐derived lactate increased H3K18la lactylation in BMSCs and upregulated the expression of target osteogenic genes. Exercise has long been reported to increase osteogenesis and attenuate osteoporosis. In this study, we found that increased lactate levels contributed to the therapeutic effects of exercise. Importantly, clinical data suggest that high lactate levels correlate with high BMD. Downregulation of H3K18la lactylation and its target osteogenic genes was observed in BMSCs isolated from patients with osteoporosis. **Figure** **9** presents the mechanism by which EC glycolysis controls osteogenesis and osteoporosis.

Bone angiogenesis plays an important role in bone metabolism, bone remodeling, and bone repair. Vascular endothelial growth factor (VEGF) increases bone blood‐vessel invasion and regulates growth plate morphogenesis.^[^ ^6b^ ^]^ Intramembranous ossification requires VEGF for angiogenesis and osteogenesis.^[^ ^23^ ^]^ EC‐derived Noggin contributes to bone formation, and exogenous Noggin improves the organization of bone vasculature.^[^ ^24^ ^]^ These studies highlighted the importance of angiocrine factors in osteogenesis. The present study found that the density of bone blood vessels decreased in an OVX‐induced mouse model of osteoporosis. ECs are highly glycolytic and generate most of their energy by converting glucose to lactate. We found that PKM2, a rate‐limiting glycolytic enzyme, was highly expressed in bone blood vessels, and its expression was decreased in the OVX model. Using a genetic mouse model with *Pkm2*‐specific deletion in ECs, we showed that PKM2 controls bone blood‐vessel formation, which in turn affects osteogenesis. Recent studies have revealed that endothelial Notch signaling and the zinc‐finger transcription factor ZEB1 control bone blood vessels and, by extension, osteogenesis in mice.^[^ ^24^, ^25^ ^]^ However, the mediator of the crosstalk between ECs and osteogenic cell lines has not yet been defined. Both Notch signaling and ZEB1 regulate glycolysis, indicating that these metabolites play a role in osteogenesis.^[^ ^26^ ^]^ These results suggest that glycolytic metabolites may mediate the crosstalk between ECs and osteogenic cells.

Lactate, previously regarded as a waste product of glycolysis, has received extensive attention as a regulator of multiple molecular processes.^[^ ^27^ ^]^ Lactate can function as a signaling molecule to affect behavior, shape the gut microbiome to alter thermogenesis, and promote PD‐1 expression in regulatory T cells to blunt tumor treatment.^[^ ^28^ ^]^ EC‐derived lactate controls adult hippocampal neurogenesis, muscle regeneration following ischemia, and pathological retinopathy.^[^ ^10^, ^29^ ^]^ We found that blood lactate levels were decreased in mice with EC‐specific deletion of *Pkm2* and in OVX mice. Previous studies have indicated that elevated lactate levels may protect against low BMD.^[^ ^30^ ^]^ This finding is consistent with our data, which showed that patients with osteoporosis had relatively low lactate levels. These data imply a possible role of lactate in the regulation of BMD. The in vitro data showed that lactate increased the differentiation of BMSCs into osteoblasts, further revealing the osteogenic function of lactate. Exercise delays the occurrence of osteoporosis, and the increased pressure or weight on the bone has been reported to play a role.^[^ ^21^, ^31^ ^]^ Exercise activates the mechanosensitive periarteriolar niche to promote osteogenesis.^[^ ^32^ ^]^ Mechanical stimuli promote reticulocalbin‐2 production by bone marrow macrophages to fuel osteogenesis.^[^ ^33^ ^]^ These studies have linked mechanical stimulation to the molecular biology of osteogenesis; however, the restoration of the relevant factors cannot fully rescue bone loss, suggesting the existence of other underlying mechanisms. The present study showed that, in OVX mice, exercise increased blood lactate levels and alleviated osteoporosis; furthermore, the addition of lactate alleviated osteoporosis. These results suggest that exercise‐derived lactate participates in osteogenesis, in addition to mechanical stimulation.

Histone lactylation has been identified as an epigenic modification type for regulating gene transcription.^[^ ^13a^ ^]^ Recent studies have revealed that histone lactylation occurs widely in human and mouse cells, and controls microglial function, cardiac repair, tumor progression, and inflammation.^[^ ^13^, ^16^, ^34^ ^]^ During osteoblast differentiation, histone lactylation of the mouse osteoblast precursor cell line increased, suggesting that histone lactylation may participate in osteogenesis.^[^ ^14^ ^]^ Our data showed decreased pan‐Kla and H3K18la lactylation in BMSCs isolated from PSkm2^ΔEC^ and OVX mice. BMSCs co‐cultured with BMECs^Δpkm2^ or treated with a conditioned cell culture medium from BMECs^Δpkm2^ showed reduced H3K18la lactylation. Joint CUT&Tag and RNA‐seq analyses revealed COL1A2, COMP, ENPP1, and TCF7L2 as the target genes of H3K18la lactylation; these genes belong to the category of bone mineralization. The expressions of COL1A2, COMP, ENPP1, and TCF7L2 were confirmed using ChIP‐qPCR. COL1A2 is an osteogenic marker associated with osteogenesis.^[^ ^35^ ^]^ COMP mediates bone growth by interacting with extracellular matrix protein 1.^[^ ^36^ ^]^ ENPP1 is a membrane‐bound glycoprotein that regulates bone mineralization.^[^ ^37^ ^]^ TCF7L2 is a key effector of canonical Wnt signaling, which is highly expressed in bones and regulates osteoblast function.^[^ ^38^ ^]^ These four genes contribute to osteogenesis and bone growth. Importantly, BMSCs isolated from patients with osteoporosis showed a decrease in histone H3K18la lactylation and the expression of the target genes COL1A2, COMP, ENPP1, and TCF7L2. Increasing exogenous lactate levels rescued the inhibited expression of COL1A2, COMP, ENPP1, and TCF7L2 and restored suppressed H3K18la lactylation in BMSCs isolated from *Pkm2* ^ΔEC^ mice. These results demonstrate that lactate‐derived lactylation of H3K18la in BMSCs regulated COL1A2, COMP, ENPP1, and TCF7L2 to control osteoporosis.

## 4 Conclusion

In summary, we have demonstrated that EC glycolysis controls bone blood‐vessel formation and attenuates osteoporosis through H3K18la lactylation in BMSCs. EC‐derived lactate functions as an angiocrine molecule that mediates crosstalk between ECs and BMSCs. Exercise helps partially protect against osteoporosis by increasing blood lactate levels. The present study highlights the importance of EC‐derived lactate in osteogenesis and osteoporosis and lays the foundation for the treatment of osteoporosis by targeting vascular metabolism.

## 5 Experimental Section

### Mice

The *Pkm2* flox/flox mice were purchased from Cyagen Biosciences. Cdh5‐P2A‐CreERT2 mice(Strain NO.T052686)were purchased from GemPharmatech (Nanjing, China). Both *Pkm2* flox/flox and Cdh5‐CreERT2 mice were on a C57BL/6 genetic background. To avoid recombination in the female germline, only Cdh5‐cre‐positive male mice were used for intercrosses. For constitutive Cre‐mediated recombination in ECs, 100 mg kg^−1^ tamoxifen (Sigma, T5648) in corn oil (Sigma) was intraperitoneally injected into pups every day from P10 to P14 for 5 consecutive days. Mice were genotyped by PCR performed on genomic DNA. Protocols and primer sequences are accessible upon request.

An ovariectomized mouse model was developed as described previously. Summarily, all 8 weeks old mice were randomly assigned to the OVX and Sham groups. The mice in the OVX group were subjected to bilateral OVX, while those in the Sham group had similar sizes of adipose tissue near the ovaries resected. The ovariectomy operation was performed according to a previously published procedure. Summarily, mice were anesthesized using isoflurane, hair was removed over their dorsum, and the skin was wiped with a povidone‐iodine solution by surgical staff. Then, the mice were placed in sternal recumbency and an incision was made in the mid‐dorsum. Later, operators located each ovary, gently removed the ovary using sterilized fine tweezers, checked for bleeding, and sutured the mice's wounds. Finally, the mice were placed in cleaned edges with heating pads until the mice recovered fully. For constitutive Cre‐mediated recombination in ECs in OVX or Sham mice, 100 mg kg^−1^ tamoxifen in corn oil was administered intraperitoneally to 9 weeks old mice for 5 consecutive days. All mice were then euthanized 11 weeks later.

For the mice treated with lactate, the mice were subcutaneously injected with lactate every 2 days after ovariectomy. Moreover, 1.5 g kg^−1^ lactate (Sigma) in 0.9% saline whose pH was adjusted to 7.4 was used. The control mice received equal volumes of 0.9% saline. The treatment of lactate lasted for 8 weeks after ovariectomy.

The mice were subjected to exercise at intervals as previously described.^[^ ^39^ ^]^ Summarily, each running session was 8 min long at 5 m min^−1^ on a rodent treadmill, followed by 10 sessions of high‐intensity running (12–16 m min^−1^, 5 min) with 2 min of rest. The high‐intensity interval exercise was performed for 5 consecutive days each week and lasted for 8 weeks. The rodent treadmill incline was set at 10°. During the exercise period, electrical stimuli were applied to encourage the mice to run. All mice were kept in alternate dark‐light cycles of 12 h at room temperature. All surgeries were performed under sodium pentobarbital anesthesia, and efforts were made to minimize suffering. All animal experiments were conducted in agreement with the NIH Guide for the Care and Use of Laboratory Animals, and with the approval of the Institutional Animal Care and Use Committee at Shanghai Changzheng Hospital (NFSC82172516).

### Isolation and Culture of BMECs

The isolation of BMECs was performed as previously described with minor revision. Summarily, the bone marrow was collected from the tibiae and femurs of mice using a sterile liver digestion medium (Gibco) supplemented with 0.1% DNaseI (Invitrogen). To obtain single BM cell suspension, BM was digested for 30 min at 37 °C under shaking conditions. BMECs were then sorted using CD31 MicroBeads, mouse (Miltenyi Biotec). Sorted BMECs were seeded on fibronectin (Sigma–Aldrich) coated dishes and cultured in endothelial cell medium (ECM, Sciencell) supplemented with endothelial cell growth supplement, fetal bovine serum, and antibiotic solution (Sciencell) at 37 °C 5% CO2. At first passage, BMECs were sorted using CD31 MicroBeads and plated for culture. The culture medium of BMECs was changed every other day and passaged upon confluency. Passages 2 to 4 of BMECs were used for subsequent experiments.

### CUT & Tag Analysis

Hyperactive Universal CUT&Tag Assay Kit for Illumina (Vazyme, TD903) and TruePrep Index Kit V2 for Illumina (Vazyme, TD202) were used for CUT&Tag assay. Summarily, 10^5^ cells were collected and washed once with 500 µL wash buffer, then they were bound to ConA beads for 10 min at room temperature. After that, cells were incubated with the first antibody (anti‐H3K18La, IgG for control) at 4 °C overnight. The secondary antibodies were added and incubated for 60 min at room temperature. Then cells were washed three times with DIG wash buffer and incubated with 0.04 µm pA/G–Tnp for 60 min at room temperature. Similarly, cells were washed three times with DIG 300 buffer, resuspended in fragmentation buffer, and incubated at 37 °C for 1 h. After fragmentation, proteinase K, buffer LB, and DNA extract beads were added to each sample. After incubation at 55 °C for 10 min, beads were rinsed with Buffer WA and Buffer WB. Then, DNA was eluted at room temperature for 5 min with 22 µL H2O. CUT&Tag libraries were built using TruePrep Index Kit V2 for Illumina (Vazyme, TD202) following the manufacturer's protocol.

The libraries were sequenced with an Illumina platform by Shanghai Biotechnology Corporation. The raw reads were preprocessed by filtering out adapters, short‐fragment reads, and other low‐quality reads. The reads were mapped using Bowtie (version 0.12.8) and called by MACS2 (version 2.2.7.1). The motifs were identified using HOMER software. The data were visualized using Integrative Genomics Viewer. Peaks with fold change ≥2 OR ≤1/2 and *p*‐value<0.05 would be analyzed by the R package DiffBind.

### Chip‐qPCR

ChIP was performed according to the manufacturer's instructions (The Agarose ChIP Kit, 26 156, ThermoFisher). Summarily, BMSCs were fixed with 1% formaldehyde for 10 min at room temperature and quenched with Glycine Solution (1X) for 5 min. Then, fixed cells were lysed with Lysis Buffer 1 containing protease inhibitors and digested with Micrococcal Nuclease (ChIP Grade) (10 U µL^−1^). The digested chromatin was incubated Dilution Buffer containing primary antibodies at 4 °C overnight on a vertical roller. Then, ChIP Grade Protein A/G Plus Agarose was added to each IP, the column was centrifuged, and the column was washed with Wash Buffer 1/2/3. Later, IP was eluted with 150 µL IP Elution Buffer, 6 µL of 5 m NaCl, and 2 µL of 20 mg mL^−1^ Proteinase K. Finally, DNA was obtained by DNA Binding Buffer, DNA Clean‐Up Column, and DNA Column Wash Buffer following the protocols. Purified DNA levels were quantitatively measured by real‐time PCR. The primers are listed in Table S2 (Supporting Information).

### Immunofluorescence

Femurs were collected from mice and immediately fixed in 4% PFA for 24 h. Decalcification was carried out with 0.5 m EDTA at 4 °C with constant slow shaking for 24 h. Then, the decalcified femurs were immersed in 20% sucrose and 2% PVP for 24 h. Finally, the bones were embedded and frozen in 8% gelatin using low‐profile blades. For immunostaining, bone sections were air‐dried, permeabilized for 10 min in 0.2% Triton X‐100, and blocked in 5% donkey serum at room temperature for 30 min. Blocked sections were probed with the primary antibodies diluted in 5% donkey serum in PBS for 2 h at room temperature or overnight at 4 °C. After primary antibody incubation, sections were washed with PBS three times and incubated with appropriate Alexa Fluor‐coupled secondary antibodies (1:400) for 1 h at room temperature. Nuclei were counterstained with DAPI. Sections were thoroughly washed with PBS, air‐dried, and sealed with nail polish.

### Study Population

From February 2021 to November 2022, 182 female patients aged 40–55 years who visited the outpatient department of orthopedics, Changzheng Hospital, were invited to participate in the study. The exclusion criteria included: 1) those who received therapy or medications that may affect BMD, such as hormone replacement therapy or steroid agents; and 2) those who had diseases that might affect bone metabolism, such as fractures or thyroid disorders. In total, 86 participants were included, and all the subjects provided written informed consent. The study protocol was approved by the Committees for Ethical Review of Research involving Human Subjects of Shanghai Changzheng Hospital (NFSC82172516).

### Statistical Analysis

All data were presented as the means ± standard deviations (SD) from at least three repeated experiments. Statistical analysis was performed using GraphPad Prism (GraphPad software 9.0, GraphPad, Bethesda, MD, USA). Unpaired Student's *t*‐test was used for the data of two‐group analysis, while comparisons between multiple groups were performed by one‐way analysis of variance (ANOVA). *p* values < 0.05 were considered as statistically significant (*p < 0.05; **p < 0.01; ***p < 0.001; and ****p < 0.0001).

## Conflict of Interest

The authors declare no conflicts of interest.

## Author Contributions

J.W., M.H., H.J., and J.M. contributed equally to this work. X.Z., C.W., T.L. performed funding and supervised the study; X.Z., C.W., Y.G., J.W., and M.H. designed the study; J.W., M.H., H.J., and J.M. designed the animal study; J.W., M.H., H.J., J.M., C.X., C.C., Z.Z., X.Z., J.Z., Z.T., Y.M., Z.C., T.S., and C.Z. performed data acquisition; J.W., M.H., C.X., and Y.G. performed data analysis; H.S. and C.X. provided technical support; J.W. and M.H. performed writing and preparing original draft; X.Z. and C.W. performed writing, reviewing, and editing. All authors have read and agreed to the published version of the manuscript.

## Supporting information

Supporting Information

## Data Availability

The data that support the findings of this study are available from the corresponding author upon reasonable request.
